# A review of duodenal metastases from squamous cell carcinoma of the cervix presenting as an upper gastrointestinal bleed

**DOI:** 10.1186/1477-7819-9-113

**Published:** 2011-09-29

**Authors:** Rani Kanthan, Jenna-Lynn Senger, Dana Diudea, Selliah Kanthan

**Affiliations:** 1 Department of Pathology and Laboratory Medicine, College of Medicine, University of Saskatchewan, Saskatoon, Saskatchewan, S7N 0W8, Canada; 2 Department of Surgery, College of Medicine, University of Saskatchewan, Saskatoon, Saskatchewan, S7N 0W8, Canada

**Keywords:** squamous cell carcinoma of the cervix, small bowel metastases, duodenal stricture, upper gastrointestinal bleed, histopathological diagnosis

## Abstract

Upper gastrointestinal bleeding due to duodenal metastases is extremely uncommon. Extra-pelvic spread of squamous cell carcinoma (SCC) of the cervix to the small bowel is rare with only 6 reported cases in the English literature since 1981(PubMed, Medline).

We report the case of a 49-year-old woman who presented with upper-gastrointestinal bleeding two years after the diagnosis of SCC of the cervix. At esophagogastroduodenoscopy, there was a stricture in the second part of the duodenum which was biopsied for a suspected neoplastic lesion. Histologic and immunohistochemical examination showed a malignant lesion with characteristics identical to her original tumor in the cervix confirming the duodenal metastases.

The clinical presentation of a 'malignant' upper-gastrointestinal bleed due to duodenal metastases from SCC of the cervix is unusual. Awareness of such infrequent patterns of metastases in cervical cancer confirmed by histopathological diagnosis is important for best practice therapeutic decisions in these patients.

## Introduction

Histopathology specimens of small bowel lesions are infrequently encountered in surgical pathology. Malignant diagnosis of such lesions accounts for only 0.4% of all cancers and 0.2% of cancer-related deaths [1]. Metastatic lesions are more common in the duodenum, jejunum and ileum than primary lesions. Though malignant melanoma is the most common extra-gastrointestinal primary to metastasize to the small bowel [2], intestinal metastases are common in end-stage adenocarcinomas of the pancreas, colon, or stomach by intraperitoneal seeding. Squamous cell carcinoma (SCC) of the cervix is the second most common gynecologic malignancy and the majority of patients usually die from local extension rather than distant metastases. It is exceedingly rare for SCC of the cervix to clinically present with symptoms related to small bowel metastases.

We herein report a case of duodenal metastases from SCC of the cervix confirmed by histopathological diagnosis that presented with upper gastrointestinal bleeding. In addition, we have provided a comprehensive review of all reported cases of SCC of the cervix metastasizing to the small bowel in the published English literature by a PubMed and Medline search as available since 1981.

## Case Report

**Figure 1:**
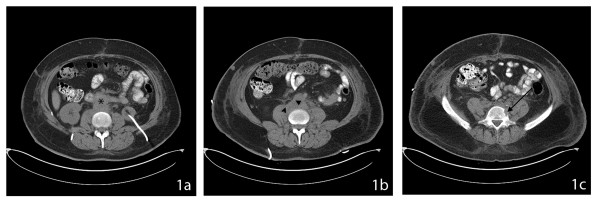
**CT Scans of the Abdomen**. **1a:**. CT scan demonstrates the presence of a soft tissue mass (*) impinging on the duodenum. **1b: **CT scan demonstrates the presence of an extensive, abnormal retroperitoneal soft-tissue mass (▲) surrounding the aorta most consistent with metastatic lympadenopathy. **1c: **CT scan also shows evidence of some lytic destruction of the left lateral aspect of the L5 vertebral body most likely related to local invasion of the metastatic disease.

A 49 year-old female presented as a surgical emergency with massive upper gastrointestinal (GI) bleeding. Her significant past history included chemotherapy and radiation treatment for invasive squamous cell carcinoma (stage IIa) of the cervix with extension to the urinary bladder two years ago. Her upper GI bleed was investigated with an emergency esophagogastroduodenoscopy. The stomach, fundus, and GI junction all appeared completely normal; however, it was difficult to advance the scope beyond the second part of the duodenum due to a stricturing abnormality. This abnormal area was suspected for an underlying neoplastic process and a tissue biopsy was obtained. While awaiting pathological confirmation of the exact nature of this neoplastic lesion, the patient was investigated with computed tomography (CT) scans as part of pre-surgical workup. The CT scans confirmed the presence of large soft-tissue mass completely encircling the abdominal aorta (▲) and impinging on the adjacent duodenum (*) as seen in Figures 1a and 1b. She was also noted to have a lytic lesion in the L5 vertebral body 1c suggesting disseminated carcinomatosis. She rapidly continued to deteriorate with decreasing urinary output and swelling of the lower legs suggesting impending renal failure and inferior vena cava compression due to nodal obstruction. Despite intensive resuscitative measures, while awaiting surgical exploration she died within 48 hours of admission. As no autopsy was conducted, the exact cause of death is unknown though it is presumed to be as a result of multiple organ failure due to disseminated carcinomatosis arising from cervical cancer.

**Figure 2:**
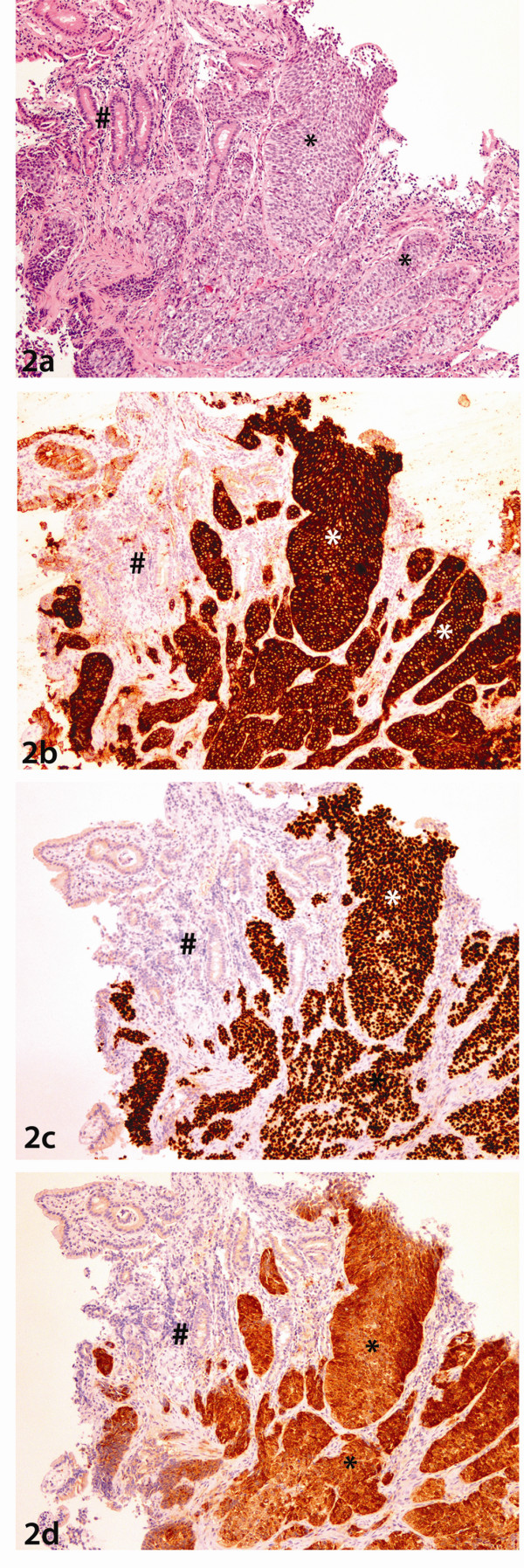
**Duodenal Biopsy**. **2a:**. Photomicrograph of haematoxylin and eosin stained slide at low power ( lens objective x2) shows the presence of cohesive sheets of malignant nonkeratinizing squamous cells (*) adjacent to normal duodenal mucosa (#) **2b: **Photomicrograph of staining with Cytokeratin 5 shows strong cytoplasmic and membrane staining of the lesional cells (*) with no staining in the adjacent duodenal mucosa (#) **2c**: Photomicrograph of staining with P63 shows strong nuclear staining of the lesional cells (*) with no staining in the adjacent duodenal mucosa (#) **2d**: Photomicrograph of staining with P16 shows diffuse cytoplasmic and nuclear staining of the lesional cells (*) with no staining in the adjacent duodenal mucosa (#)

On histopathological examination, the duodenal biopsy showed the presence of small bowel mucosa admixed with cohesive sheaths of neoplastic cells highly reminiscent of malignant nonkeratinizing squamous cell carcinoma (Figure 2a). In this context, additional immunohistochemical analysis showed the lesional cells to be strongly positive with staining to antibodies of CK5, p63 and p16 (Figures 2b, 2c, 2d). Good internal control of negative staining in the adjacent duodenal mucosa was observed.

**Figure 3:**
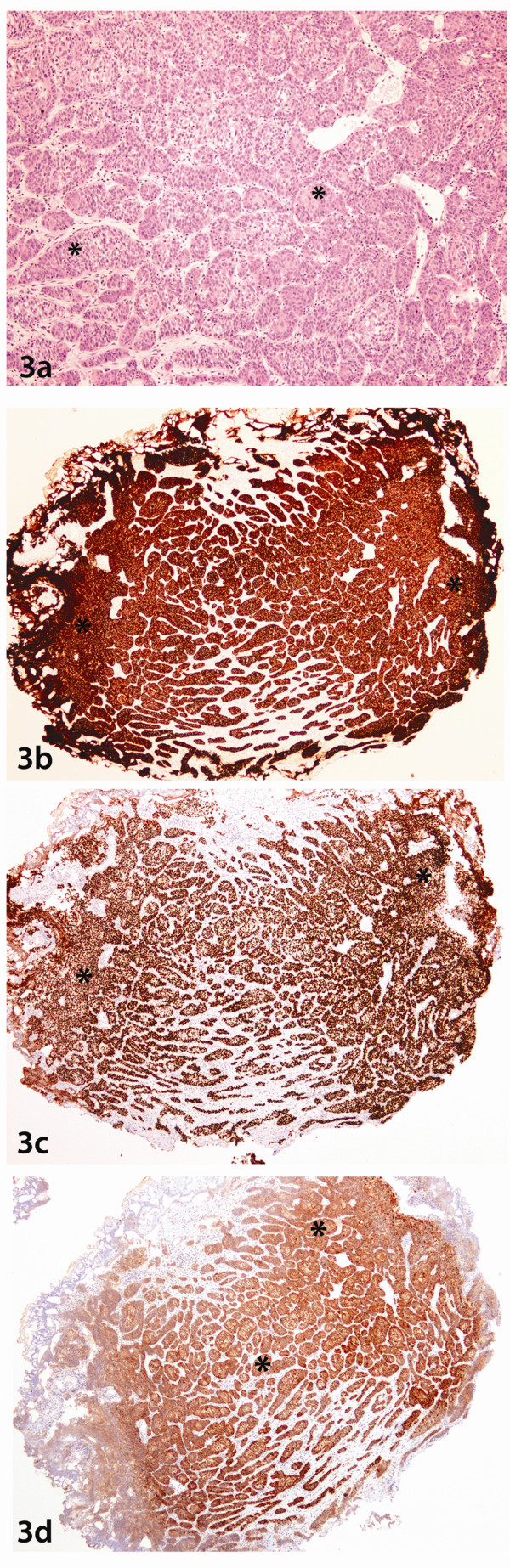
**Bladder Biopsy**. **3a:**. Photomicrograph of haematoxylin and eosin stained slide at low power ( lens objective x2) shows the presence of cohesive sheets of malignant nonkeratinizing squamous cells(*) similar to the cells seen in Figure 2a(*). **3b**: Photomicrograph of staining with Cytokeratin 5 shows strong cytoplasmic and membrane staining of the lesional cells (*) as seen in Figure 2b. **3c**: Photomicrograph of staining with P63 shows strong nuclear staining of the cells (*) as seen in the duodenal biopsy in Figure 2c. **3d**: Photomicrograph of staining with P16 shows diffuse cytoplasmic and nuclear staining of the lesional cells (*) similar to those seen in Figure 2d.

**Figure 4:**
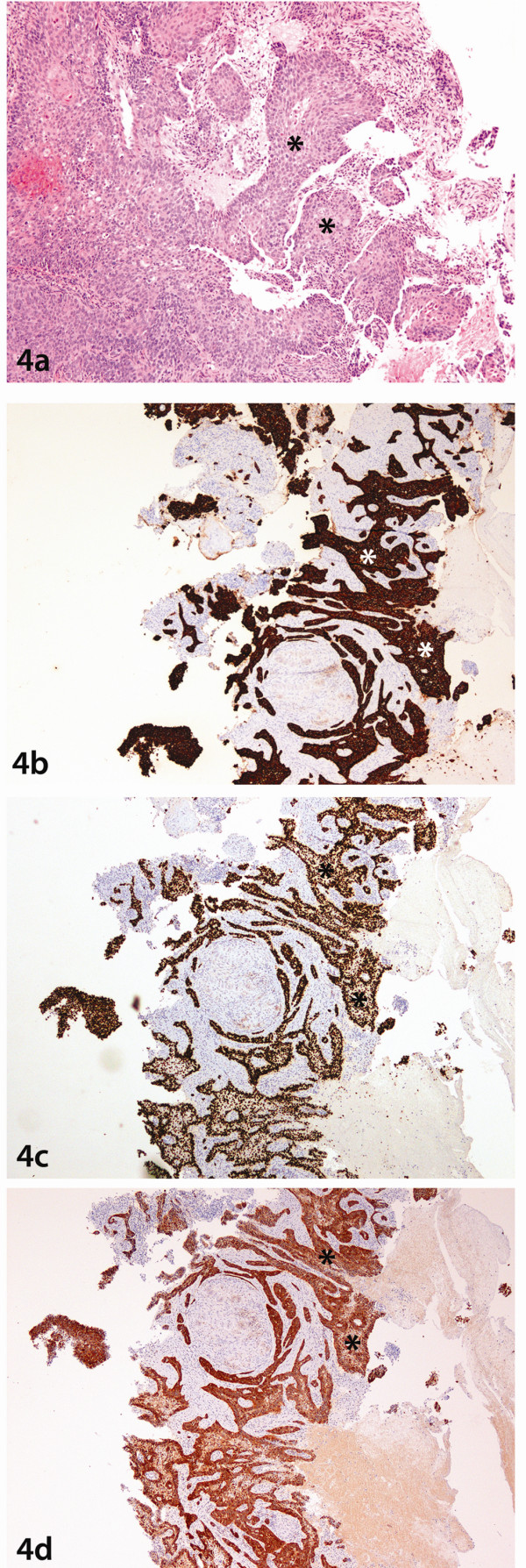
**Cervix Biopsy**. **4a:**. Photomicrograph of haematoxylin and eosin stained slide at low power (lens objective x2) shows the presence of cohesive sheets of malignant nonkeratinizing squamous cells(*) similar to the cells seen in Figure 2a (*). **4b: **Photomicrograph of staining with Cytokeratin 5 shows strong cytoplasmic and membrane staining of the lesional cells (*) as seen in Figure 2b. **4c: **Photomicrograph of staining with P63 shows strong nuclear staining of the cells (*) as seen in the duodenal biopsy in Figure 2c. **4d: **Photomicrograph of staining with P16 shows diffuse cytoplasmic and nuclear staining of the lesional cells (*) similar to those seen in Figure 2d.

In order to confirm the metastatic nature of these cells, the previous histological slides of the bladder (Figure 3a) and cervix biopsies (Figure 4a) were reviewed which confirmed their histomorphological similarity to the neoplastic cells in the current duodenal sample. Immunohistochemical staining patterns of the previous lesions in the bladder (Figures 3b, 3c, 3d), and cervix (Figures 4b, 4c, 4d) were also identical to the current indexed duodenal biopsies (Figures 2b, 2c, 2d). The diagnosis of metastatic squamous cell carcinoma of the cervix to the duodenum was pathologically confirmed.

## Review and Discussion

Using PubMed and Medline, a comprehensive literature search limited to the English language was performed using the text words "duodenum", "jejunum", "ileum", "small bowel" or "small intestine" initially with "squamous cell carcinoma" and "cervix" and repeated with "carcinoma cervix", "uterine carcinoma" and "cervical neoplasia". Reference lists of articles identified by this strategy were searched with the selection of additional relevant publications for review and analysis. Table 1 provides a comprehensive review of the published cases of small bowel metastases from squamous cell carcinoma (SCC) of the cervix in the English literature since 1981 including details of : i) reference #, ii) author, iii) age, iv) time interval to metastases, v) presenting symptoms, vi) site of metastasis, vii) confirmation of diagnosis viii) treatment, and ix) outcome [3-9].

**Table 1:** Small Bowel Metastases from Squamous Cell Carcinoma of the Cervix as Reported in the Literature (PubMed and Medline available since 1981) - search terms: "squamous cell carcinoma" AND "cervix"/"carcinoma cervix" with "duodenum", "jejunum", "ileum", "small bowel" and/or "small intestine"

Ref #	Authors	Age	Stage of SCC of cervix at diagnosis	Presenting Symptoms	Site of Metastasis	Confirmation of Diagnosis	Time interval to metastases	Treatment	Outcome
3	Gurian, 1981	64	IIIb	Hematocrit 15% (occult bleeding)	1^st ^part duodenum (gastric outlet)	Biopsy of duodenal lesion	Synchronous metastases	Refused surgical intervention	Death

4	Misonou, 1988	69	Ia	Small intestinal perforation with panperitonitis	Multiple mets to the small intestine -Jejunum	Jejunal resection -histopathology	13 years	Laparotomy	Death

5	Christopherson 1985	42	IIIb	1 month h/o intermittent nausea and vomiting, upper abdominal pain (small bowel obstruction)	Single mass-stomach, ileum, omentum, transverse colon	Histopathology of resected 10 × 10 cm mass	2 years	Laparotomy, chemotherapy	Recovery

6	Hulecki, 1985	48	Ib	Gross hematuria from the conduit	Ileal conduit	Biopsy of 1 × 1 cm mass about 10 cms from the stoma	7 years	Laparotomy	Recovery

7	Mathur, 1984	35	NR	Abdominal Pain, persistent vomiting, constipation, (isolated stricture ileum)	Ileum 5 cm proximal to ileo-caecal junction	Histopathology of Rt.Hemicolectomy	Synchronous metastases	Right Hemicolectomy	Recovery

8	Lee, 2011	50	IIa	Epigastric pain	2^nd ^part duodenum	Multiple biopsies Ampulla of Vater	2 years	Chemotherapy	NR

9	Ewing, 1981*	*61*	*IIa*	*Nausea, vomiting, abdominal distension, 50 lb weight loss*	*Paraaortic LN causing high level duodenal obstruction*	*Histopathology of paraaortic node*	*9 years*	*Laparotomy with bypass surgery*	*Died 12 weeks post-op*

	***Kanthan 2011***	***49***	***IIa***	***Upper gastrointestinal bleeding***	***2***^***nd ***^***part of duodenum***	***Biopsy of duodenal lesion***	***2 years***	***Died within 48 hours of admission***	

NR -Not reported

NA-Not available

Squamous cell carcinoma (SCC) of the cervix is the second most common gynecologic malignancy [3,10]. In the cervix, SCC accounts for 80-85% of all cases, with 15-20% being adenocarcinomas [10]. This common neoplasm may occur at any stage of life; however, it is most commonly diagnosed in the fifth decade, with nearly half of the cases being diagnosed before the age of 35 [11]. The decreasing age of occurrence is attributed to the accepted social norms of earlier onset of sexual activity and complemented with earlier detection by active screening programs. In North America, as a result of the implementation of active screening programs, approximately 60% of cases are identified at Stage I, with 25%, 10% and 5% detected in stages II, III and IV respectively [11]. Most cases with Stage IV disease do not die due to distant metastases, but rather as a result of local extension into and around the urinary bladder, causing ureteral obstruction, pyelonephritis and uremia as seen in our index case who also had distant metastases to the duodenum.

Carcinoma of the cervix usually spreads in an orderly and predictable fashion [5]. The earliest and most common metastases are by direct extension to the contiguous structures including the vagina, peritoneum, urinary bladder, ureters, rectum and paracervical tissue [5]; however, distant metastatic spread with unusual patterns such as pulmonary lymphangitic carcinomatosis have also been reported [12]. Up to 50% of Stage IV patients can present with distant metastases [12]. Common sites of such occurrences are the liver, lungs, and bone marrow. The gastrointestinal tract is involved in approximately 8% of patients with carcinoma of the cervix; these being commonly found in the rectosigmoid as a result of local extension [3]. Gastric lesions are identified in less than 2% of patients with carcinoma of the cervix, and are usually asymptomatic [3]. Isolated metastases to the small bowel are exceedingly rare. Such spread is believed to occur commonly through the lymphatics, usually the para-aortic (as presumed in our case) or mesenteric nodes to the bowel's serosa and less often via the blood stream or by peritoneal seedlings [3,4,7].

Neoplasms in the small bowel are rare, with a global incidence of less than 1.0 per 100 000 people [1]. In the USA only 0.4% of all cancers are in the small bowel, making up 0.2% of all cancer-related deaths [1]. Malignant tumors of the small bowel are unusual, and account for only 1-5% of all GI tract malignancies [13]. Metastatic lesions to the small bowel are more common than primary lesions, and most commonly arise from malignant melanoma, carcinoma of the lung, genitourinary cancers, breast cancer, Kaposi's sarcoma, colonic and renal cell carcinomas [2,14]. Adenocarcinoma is estimated to account for 35-50% of small bowel tumors, with up to 20-40% being carcinoids and 14% being lymphomas [13]. Interestingly, the distribution of the metastases in the small bowel correlates with the histopathological tumour subtype: adenocarcinomas are predominantly located in the duodenum and proximal jejunum while lymphomas and carcinoids are more frequently located in the jejunum or ileum while sarcomas are seen through the entire small bowel [13,14]. The most common presenting symptom of small bowel lesions is a partial or complete bowel obstruction and less commonly, bowel perforation, persistent abdominal pain, or hemorrhage(overt or occult) [3,7]. Malignancy only accounts for 1-4% of upper GI bleeds as hemorrhage is more common in benign tumors [14].

It remains puzzling why small-bowel metastases are such rare events, even though anatomically the small intestine makes up 75% of the length and 90% of the absorptive surface of the esophagogastrointestinal system [1,13]. Several mechanisms have been identified by which metastases to the small bowel can occur. These include peritoneal dissemination, direct spread from an intra-abdominal malignancy, haematogenous and by lymphatic spread [3,4,7]. Recognition of the paucity of neoplasms metastasizing to the small bowel has prompted many theories of antitumor mechanisms that may be involved in the innate local micro-environmental protection in the small intestine [1,13-16]. These theories include:

### A. Motility and Rapid Transit Time

Unlike the solid feces passing through the colon, chyme of the small intestine is liquefied, causing less mucosal irritation and reducing mechanical trauma. Additionally, transit through the small intestine is more rapid than through the large intestine, thereby shortening the exposure of potential carcinogens in the chyme to the mucosal surface.

### B. Immune Features

Immune protection is abundant through the small intestine, with numerous lymphoid cells in the mucosa and submucosa. Further, the small bowel is responsible for large secretions of IgA, an antibody intimately involved in mucosal protection. Compared to the rest of the population, patients on immunosuppressive agents have an increased risk of tumorigenesis in the small bowel. Additionally, immunological abnormalities such as IgA deficiency, Crohn's and celiac disease have a greater propensity for the development of small bowel tumors.

### C. Intraluminal Microbial Ecosystem

As the bacterial counts in the small bowel are absent or considerably lower than the large bowel, there is minimal exposure to the potentially carcinogenic chemical products of bacterial breakdown. Additionally, the relative intraluminal alkalinity of the small bowel can prevent the formation of potentially carcinogenic nitrosamines. Furthermore, the small bowel contains benzopyrene hydroxylase and other tumor-inhibiting components that may aid in neutralizing carcinogens.

### D. Intraluminal environment

It is estimated that every sixteen minutes, one gram of small intestinal mucosa is replaced, and the entire mucosal layer including the absorptive, glandular, and neuroendocrine cells is restored every 4-7 days. Such high turnaround of mucosal cells is probably incompatible with the "critical cell mass" required for tumorigenesis. Liquefied chyme in the small bowel may act as a mucosal barrier to potential carcinogens. Additionally, small bowel stem cells are well protected as they are buried deep within the crypts.

A detailed review of the published English literature yielded only six cases of squamous cell carcinoma of the cervix with documented metastases to the small bowel (Table 1). The most common site of metastases was the ileum (3 cases) followed by the duodenum (2 cases) and the jejunum (1 case). Indirect small bowel involvement was also noted by Ewing et al, who reported metastases to the paraaortic lymph nodes causing a high level complete duodenal obstruction with massive gastric dilatation caused by recurrent cervical cancer [9]. Varied times between the primary and the manifestation of metastatic lesions is reported in the literature ranging from being synchronous [3,7] to metachronous with a delayed time interval ranging from 2-13 years [4,5]. Clinical presentations reported, though varied, do not describe overt upper gastrointestinal bleed as seen in our index case. The overall long term prognosis of cases with duodenal metastases is extremely poor as it probably indicates disseminated disease. This is further compounded by delayed diagnosis of these unusual lesions [2,14].

## Conclusions

In conclusion, duodenal metastasis from SCC of the cervix is an extremely uncommon 'malignant' cause of upper gastrointestinal bleeding. Yet, accurate recognition of such unusual patterns of metastases in cervical cancer by histopathology is vital for best practice therapeutic decisions in these patients.

## Conflict of interest

The authors declare no conflicts of interest.

## Authors' contributions

All authors participated fully in the conception, development, and creation of this manuscript. All authors read and approved the final version of the manuscript.
